# Investigating Different DNA Methylation Patterns at the Resolution of Methylation Haplotypes

**DOI:** 10.3389/fgene.2021.697279

**Published:** 2021-06-28

**Authors:** Xiaoqing Peng, Yiming Li, Xiangyan Kong, Xiaoshu Zhu, Xiaojun Ding

**Affiliations:** ^1^Center for Medical Genetics & Hunan Key Laboratory of Medical Genetics, School of Life Sciences, Central South University, Changsha, China; ^2^School of Computer Science and Engineering, Central South University, Changsha, China; ^3^School of Computer Science and Engineering, Yulin Normal University, Yulin, China

**Keywords:** methylation haplotype, differentially methylated region, cell differentiation, homologous chromosomes, methylation consistency, hypomethylation consistency

## Abstract

Different DNA methylation patterns presented on different tissues or cell types are considered as one of the main reasons accounting for the tissue-specific gene expressions. In recent years, many methods have been proposed to identify differentially methylated regions (DMRs) based on the mixture of methylation signals from homologous chromosomes. To investigate the possible influence of homologous chromosomes on methylation analysis, this paper proposed a method (MHap) to construct methylation haplotypes for homologous chromosomes in CpG dense regions. Through comparing the methylation consistency between homologous chromosomes in different cell types, it can be found that majority of paired methylation haplotypes derived from homologous chromosomes are consistent, while a lower methylation consistency was observed in the breast cancer sample. It also can be observed that the hypomethylation consistency of differentiated cells is higher than that of the corresponding undifferentiated stem cells. Furthermore, based on the methylation haplotypes constructed on homologous chromosomes, a method (MHap_DMR) is developed to identify DMRs between differentiated cells and the corresponding undifferentiated stem cells, or between the breast cancer sample and the normal breast sample. Through comparing the methylation haplotype modes of DMRs in two cell types, the DNA methylation changing directions of homologous chromosomes in cell differentiation and cancerization can be revealed. The code is available at: https://github.com/xqpeng/MHap_DMR.

## 1. Introduction

In recent years, the revealing of the mechanisms behind the diseases has been performed from different angles, such as mutated genes, altered DNA methylation (Eden et al., 2003; Baylin, 2005), non-coding RNAs (Yan et al., 2017, 2018; Chen et al., 2019; Lan et al., 2020), microbes (Yan et al., 2019, 2021), etc. Differentially methylated regions (DMRs) are the main explanation accounting for the diversity of gene expression in different cell types in a body. Differentiation-associated differential methylation profiles were observed on cell types under different stages of development and differentiation (Laurent et al., 2010). Recent studies show that altered DNA methylation has a very close relationship with diseases. In cancer genomes, the promoter regions of tumor suppressor genes are altered to be hypermethylated (Baylin, 2005), while the promoter regions of tumor genes are altered to be hypomethylated (Eden et al., 2003). Identifying DMRs can promote revealing the mechanisms in tissue-specific/diseases-specific gene expression (Scott et al., 2020) and tissue-specific DMRs can be used as feature markers in identifying the tissues-of-origin of cfDNAs in noninvasive diagnosis (Hu et al., 2019; Xiaoqing et al., 2020).

Infinium HumanMethylation450 BeadChip and Infinium MethylationEPIC BeadChip provide a convenient way to measure the methylation levels of CpG sites in CpG islands and gene regions. In BreadChips, the methylation level of a certain CpG site is estimated by using the ratio of intensities between methylated and unmethylated alleles. In recent years, due to the development of sequencing technology, bisulfite sequencing (BS-Seq) makes to reveal the methylation status of each cytosine site on a read become possible. The numbers of methylated cytosines and unmethylated ones of each cytosine site can be measured, respectively. Recently, by using deep-learning, DNA methylation status of each cytosine site can be deduced from Nanopore sequencing reads (Ni et al., 2019). In both BeadChip and BS-Seq, molecules derived from two homologous chromosomes are not discriminated and are always processed together.

Based on the methylation profiles extracted from BeadChips or BS-Seq data, many methods have been proposed to identify DMRs in different tissues or cell types. These methods can be roughly divided into two categories: differentially methylated cytosine site (DMC)-based methods and candidate region-based methods. In DMC based methods, methylation levels of CpG sites can be calculated based the raw methylation information of CpG sites (Catoni et al., 2018; Condon et al., 2018; Xu et al., 2020), estimated by beta-binomial distribution considering the biological variances and sample variances (Feng et al., 2014; Park et al., 2014; Lea et al., 2015; Wu et al., 2015; Park and Wu, 2016; Wen et al., 2016) or estimated by considering the spatial correlation (Hansen et al., 2012; Hebestreit et al., 2013; Wu et al., 2015; Sun and Yu, 2016). Then, DMCs are identified and DMRs are formed by merging the neighboring DMCs satisfying some defined criteria, such as DMCs with *p*-values less than a certain threshold, the distance between the DMCs less than a cutoff value, and the minimum number of DMCs required in a region.

In candidate region-based methods, there are two types of candidate regions, including sample-dependent candidate regions and sample-independent ones. The sample-independent candidate regions are predefined on the genome with a fixed-size or sliding window (Stockwell et al., 2014; Wang et al., 2015; Catoni et al., 2018). The sample-dependent candidate regions are generated according to the coverage, the depth of CpG sites, the methylation levels of CpG sites in samples, and the methylation changes of CpG sites among multi-samples. Then DMRs are identified by comparing the methylation of regions among different samples (Su et al., 2012; Lokk et al., 2014; Liu et al., 2015; Jühling et al., 2016; Gomez et al., 2019).

As we known, the allele-specific methylation is a special phenomenon of DNA methylation, which is that the methylation of an allele on two homologous chromosomes is not consistent. With the development of high-throughput sequencing technology, the region capture based sequencing and the genome-wide sequencing have been widely used for detecting allele-specific methylation sites. Some strategies and algorithms also contribute to improve the identification of allele-specific methylation (Cheung et al., 2017; Abante et al., 2020). However, the research on identifying allele-specific methylation is limited to the alleles, and the influence of homologous chromosomes on methylation analysis should be investigated genome wide.

In the methods of identifying DMRs, the reads from homologous chromosomes are processed together, and the methylation levels of CpG sites are calculated based on the mixture of reads from homologous chromosomes. The influence of homologous chromosomes on methylation analysis was not considered and investigated. To investigate the possible influence of homologous chromosomes on methylation analysis, we construct methylation haplotypes for homologous chromosomes on the sample-independent candidate regions. Then the methylation consistency of paired methylation haplotypes from homologous chromosomes in different cell types is compared. Further, DMRs are identified at the resolution of methylation haplotypes. The proposed method in this paper not only can be applied to methylation analysis, but also can provide a clear explanation for the methylation difference at the resolution of methylation haplotypes.

## 2. Materials and Methods

**Figure 1:**
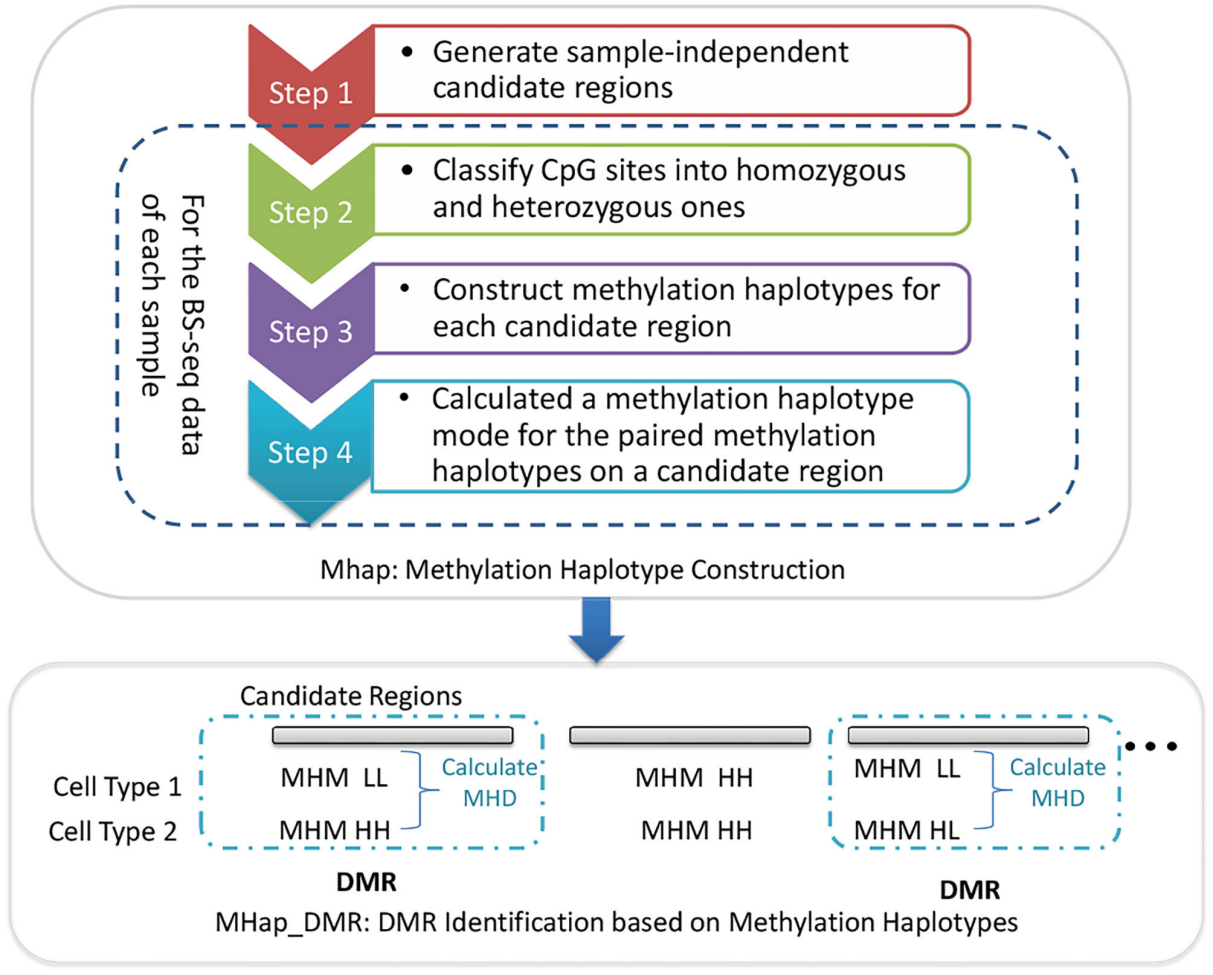
The framework of MHap and MHap_DMR.

In this paper, two methods, MHap and MHap_DMR, are proposed to construct methylation haplotypes and identify DMRs based on methylation haplotypes, respectively. MHap is a method for constructing methylation haplotypes, which consists of four steps. Firstly, it generates sample-independent candidate regions based on genomic information, such as CpG islands and CpG dense regions. Then, for the BS-seq data of each sample, it classifies CpG sites into homozygous and heterozygous ones, and then constructs methylation haplotypes for each candidate region. Finally, the paired methylation haplotypes of homologous chromosomes are represented by methylation haplotype modes (MHMs). MHap_DMR is the method designed to identify DMRs based on methylation haplotypes. The framework of MHap and MHap_DMR is shown in Figure 1 and the detail of each step in the proposed methods will be described in the following subsections.

### 2.1. Materials

To investigate the influence of homologous chromosomes on methylation analysis, 12 WGBS datasets of 10 different tissues/cell types are involved in this study, including two lower leg skin samples and two tibial nerver samples downloaded from the ENCODE project (The ENCODE Project Consortium, 2012) (access sample id: ENCSR930WUY, ENCSR128RMY, ENCSR752OCM, and ENCSR658MZU), breast cancer sample and normal breast sample in the GEO database under the accession number GSE29069 (Hon et al., 2012), adipose-derived stem (ADS) cells and mature fat cells (adipocytes derived from the ADS cells) in the NCBI SRA database under the accession number SRA023829.2 (Lister et al., 2011), embryonic stem cells (hESCs) and foreskin fibroblasts (hESC-Fibro cells) in the GEO database under the accession number GSE19418 (Laurent et al., 2010), mature B cells and hematopoietic stem cells in the GEO database under the accession number GSE31971 (Hodges et al., 2011). The WGBS datasets were aligned to the human reference genome (hg38) and the methylation statuses of cytosines on reads were called by using Bismark (Krueger and Andrews, 2011).

### 2.2. MHap: Methylation Haplotype Construction

Due to the limited read lengths and the uneven distribution of CpG sites, it is challenging to construct two complete methylation haplotypes for two homologous chromosomes. Thus, sample-independent candidate regions are predefined on CpG dense regions, and methylation haplotypes are constructed for homologous chromosomes in these regions. MHap is proposed to construct methylation haplotypes for homologous chromosomes based on the overlapping methylation statuses of heterozygous methylated CpG sites on reads. The details of MHap is described as following.

#### 2.2.1. Generate Sample-Independent Candidate Regions

MHap generates sample-independent candidate regions based on the CpG island information and the distance between neighboring CpG sites. In order not to hide local methylation signals, CpG islands are usually divided into a number of candidate regions, each of which contains at least 7 CpG sites. For other regions with densely located CpG sites, a distance-based clustering algorithm is applied to generating candidate regions, which contains at least 7 CpG sites also and the distances between neighboring CpG sites are not >20 bp. As shown in Table 1, for each chromosome, the number of candidate regions and the corresponding averages of CpG numbers and region lengths are listed. Then, MHap will construct methylation haplotypes for homologous chromosomes on these candidate regions. of the candidate regions.

**Table 1:** The number, the average number of CpGs, and the length of candidate regions in each chromosome.

**Chromosome**	**Num. of**	**Ave. Num. of**	**Ave. length of**
	**candidate regions**	**CpGs**	**candidate regions**
chr1	26,643	10.48	92.11
chr2	20,446	10.46	91.71
chr3	14,013	10.46	93.08
chr4	13,316	10.49	95.06
chr5	14,411	10.46	93.70
chr6	14,378	10.49	93.86
chr7	16,809	10.42	92.33
chr8	12,429	10.43	92.64
chr9	13,798	10.44	91.80
chr10	13,482	10.46	91.90
chr11	14,194	10.43	91.38
chr12	13,107	10.42	93.56
chr13	7,503	10.41	93.78
chr14	9,217	10.44	91.07
chr15	9,173	10.49	89.84
chr16	14,837	10.36	91.21
chr17	17,285	10.45	92.24
chr18	6,419	10.55	92.72
chr19	20,663	10.51	95.05
chr20	8,844	10.40	90.39
chr21	5,597	10.70	92.66
chr22	8,153	10.38	87.39
chrX	10,687	10.42	97.36
chrY	1,982	10.31	103.24

#### 2.2.2. Classify CpG Sites Into Homozygous and Heterozygous Ones

**Figure 2:**
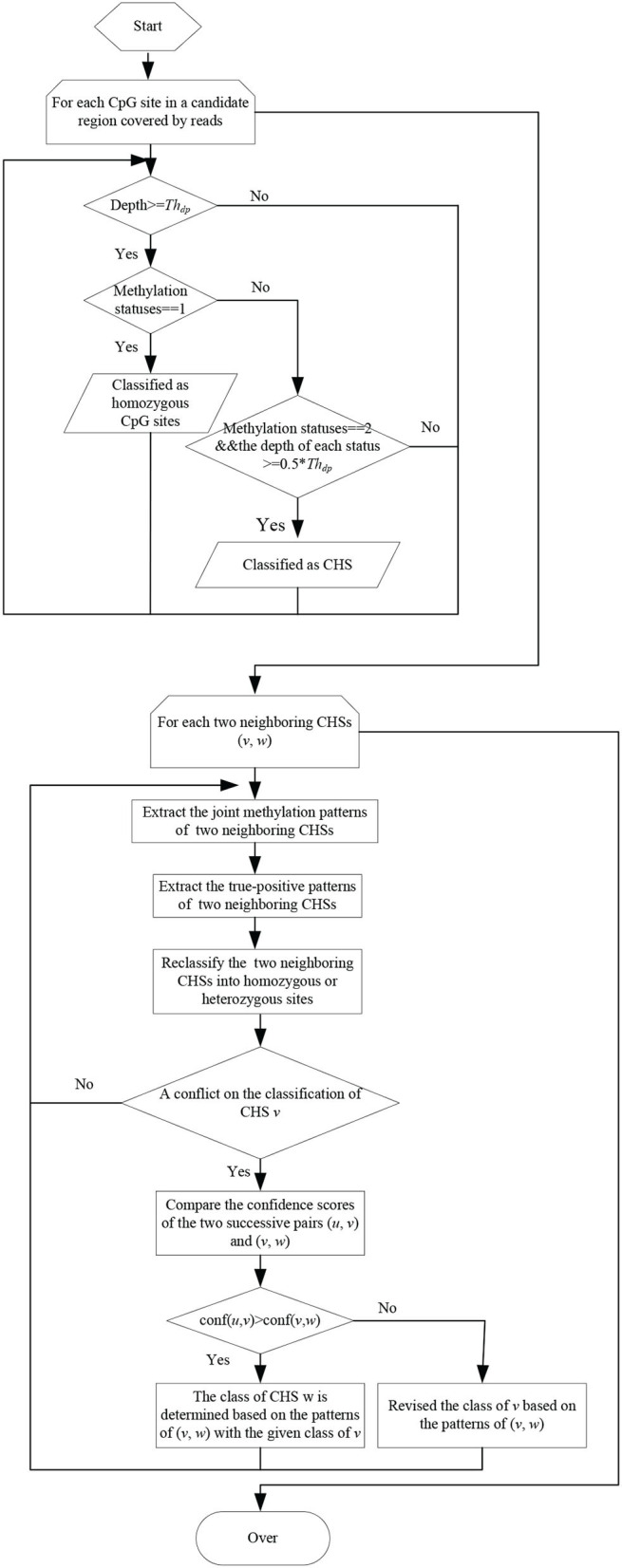
The flowchart of classifying CpG sites into homozygous and heterozygous ones.

The flow char of classifying CpG sites into homozygous and heterozygous ones is illustrated as in Figure 2. For each sample, the reads falling in candidate regions are collected. In these candidate regions, firstly, CpG sites with depth less than a threshold *Th*_*dp*_ are filtered out. Then the remaining CpG sites are classified into homozygous sites and candidate heterozygous sites(CHSs) based on the types of methylation statuses and the corresponding depths. If a CpG site has only one methylation status with depth not less than *Th*_*dp*_, it is considered as a homozygous site. If it has two methylation statuses and the depth of each status is not less than half of *Th*_*dp*_, it is considered as a CHS.

Due to the sequencing errors and the bisulfite conversion rates, the identified CHSs inevitably contain false-positives. The joint methylation statuses of neighboring CHSs on the same reads can help to distinguish true-positives from false-positives. Thus, the joint methylation statuses of two neighboring CHSs on the covering reads are extracted and can be represented as 00/11/01/10 patterns. In MHap, the frequency of each pattern on two neighboring CHSs is calculated, and patterns with frequency <2 are filtered. Then, one or two true-positive patterns are identified according to the ratios of the corresponding frequencies to the total frequency of all patterns or to the maximum frequency. If there is a pattern with the maximum frequency among other patterns and the ratio of its frequency to the total frequency of all patterns is above a threshold (recommended as 0.6), it is considered as the only one true-positive pattern on the two neighboring CHSs. Otherwise, if there are two patterns with higher frequencies than other patterns and the ratio of the second maximum frequency to the first maximum frequency is not less than a threshold (recommended as 0.4), it is considered that there are two true-positive patterns on the two neighboring CHSs. Then two neighboring CHSs are reclassified into homozygous or heterozygous ones based on the true-positive patterns.

Pairs of neighboring CHSs are processed sequentially. Assume there are three successive CHSs (*u, v, w*). During the processing of two successive pairs (*u, v*) and (*v, w*), the unbalance join depths may result in a conflict on the classification of the overlapped CHS *v*. To handle with this conflict, a confidence score is calculated for each pair of neighboring CHSs, computed as the ratio of the total frequency of true-positive patterns on two sites to the maximum depth among three CpG sites, as defined in Equation (1). If *conf*(*u, v*)> = *conf*(*v, w*), the class of *v* will be not changed, and the class of *w* will be determined based on the joint methylation statuses of (*v, w*) with the given class of *v*. If *conf*(*u, v*) < *conf*(*v, w*), the class of *v* will be revised based on the true-positive patterns of (*v, w*).

(1) $$conf(u,v)=\frac{\sum_{p}^{p\in TP}f(p)}{\max(d(u),d(v),d(w))}$$

where *TP* denotes the set of true-positive patterns of (*u, v*), *f*(*p*) denotes the frequency of pattern *p*, and *d*(*u*), *d*(*v*), and *d*(*w*) denote the depths of *u*, *v*, and *w*, respectively.

#### 2.2.3. Construct Methylation Haplotypes for Each Candidate Region

After classifying CpG sites into homozygous and heterozygous ones, the skeletons of two methylation haplotypes are constructed by linking the patterns of neighboring heterozygous sites sequentially. Then, a pair of methylation haplotypes are constructed by padding the homozygous CpG sites into the skeletons.

#### 2.2.4. Definition of Methylation Haplotype Mode

Each methylation haplotype can be represented by a 0–1 string. To simplify the comparison between methylation haplotypes, each methylation haplotype is converted into a label based on its 0–1 string, defined in Equation (2). Then, two labels of the paired methylation haplotypes on a candidate region, denoted as *LL*, *HL*, *LN*, *LM*, *NN*, *MM*, *MN*, *HN*, *HM* or *HH*, are termed as a methylation haplotype mode (MHM).

(2) $$Label(s)=\{\begin{array}{l} L,\text{if}MH(s)\leq 0.25\\ N,\text{elseif}MH(s)\leq 0.5\\ M,\text{elseif}MH(s)\leq 0.75\\ H,\text{else} \end{array}$$

where $MH(s)=\frac{\sum_{i=1}^{len(s)}\left(s_{i}-0\right)}{len(s)}$, *s* represents the 0–1 string of a methylation haplotype, *len*(*s*) represents the length of *s*, and *s*_*i*_ is the *i*-th character in *s*.

### 2.3. Map_DMR: DMR Identification Based on Methylation Haplotypes

Based on the MHMs of each candidate region among different samples, MHap_DMR identifies DMRs by comparing the MHMs directly. If the MHMs are identical, the candidate region is considered as a non-DMR. Otherwise, a methylation haplotype difference (MHD) between a pair of samples or groups is calculated, defined as in Equation (3). Then, the methylation difference among multi groups on the region can be defined as the maximum MHD among pairs of groups.

(3) $$\begin{array}{l} MHD(g_{i},g_{j})=\max(abs(MH(g_{i1})-MH(g_{j1})),abs(MH(g_{i2})\\ \;-MH(g_{j2}))) \end{array}$$

where *g*_*i*_ and *g*_*j*_ denote group *i* and *j*, *g*_*i*1_ and *g*_*j*1_ denote the 0–1 strings of methylation haplotypes with higher *MH* values in *g*_*i*_ and *g*_*j*_, respectively, and *g*_*i*2_ and *g*_*j*2_ denote the 0–1 strings of methylation haplotypes with lower *MH* values in *g*_*i*_ and *g*_*j*_, respectively.

To investigate the influence of homologous chromosomes on methylation analysis, we applied MHap to construct methylation haplotypes for 12 WGBS datasets of 10 different tissues/cell types. MHap constructs methylation haplotypes for each sample based on the alignment file and candidate regions. Methylation haplotypes covering more than 3 CpG sites are defined as valid methylation haplotypes (VMHs). Table 2 lists the number of candidate regions with VMHs contained by each sample. It can be observed that the average number of CpG sites in these candidate regions is >10, and the average number of covered CpG sites in VMHs is ranging from 5.9 to 8.9.

**Table 2:** Statistics of candidate regions with methylation haplotypes in different samples.

	**Num. of**	**Ave. Num. of**	**Ave. Num. of**
**Sample**	**candidate regions**	**CpG sites**	**covered CpG**
	**with VMHs**	**in candidate regions**	**sites in VMHs**
Mature fat cells	249,253	10.51	5.91
Adipose-derived stem cells	256,671	10.51	6.08
Breast cancer sample	233,973	10.49	6.69
Normal breast sample	223,692	10.49	6.22
Hematopoietic stem cells	172,536	10.44	6.38
Mature B cells	138,053	10.44	5.20
Embryonic stem cells	220,970	10.63	6.05
Foreskin fibroblasts	213,317	10.66	6.10
Lower_leg_skin_1	228,263	10.30	8.67
Lower_leg_skin_2	244,369	10.36	8.96
Tibial_nerve_1	239,034	10.36	8.81
Tibial_nerve_2	225,728	10.31	8.67

## 3. Result

### 3.1. Majority of Methylation Haplotypes Are Consistent Between Homologous Chromosomes

MHMs *HH* and *LL* denote that the paired methylation haplotypes of two homologous chromosomes are simultaneously hypermethylated (*HH*) or hypomethylated (*LL*). Both the *HH* and *LL* are considered as consistent MHMs. Then, the methylation consistency between two homologous chromosomes in a sample can be defined as the ratio of the number of CpGs in VMHs with consistent MHMs to that in all VMHs.

**Figure 3:**
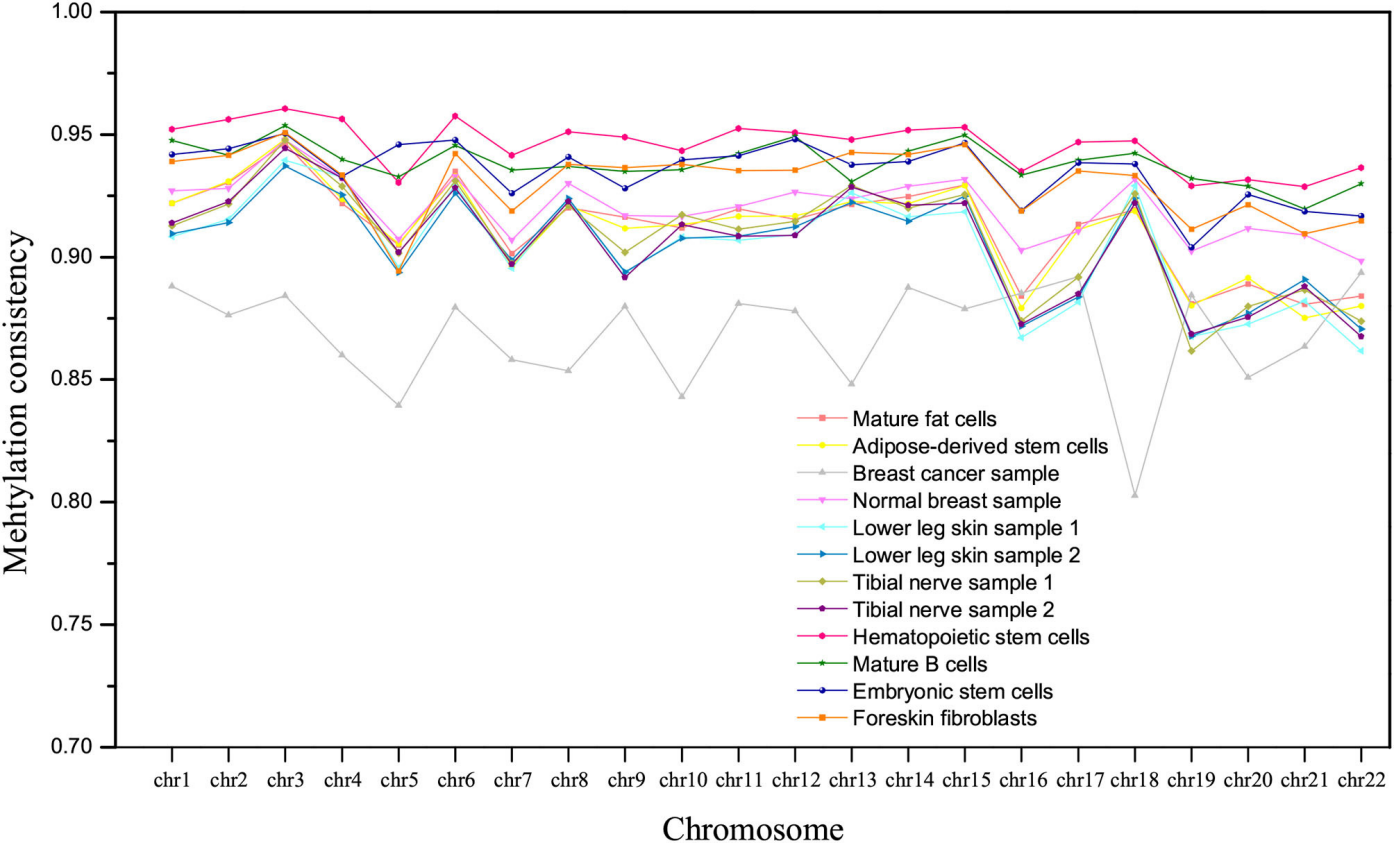
The comparison of methylation consistency of homologous chromosomes in different tissues/cell types.

The methylation consistency of homologous autosomes in different tissues/cell types is compared, as shown in Figure 3. For normal tissues or cell types, the methylation consistency is above 90% on average, especially in hematopoietic stem cells. A lower methylation consistency can be observed in the breast cancer sample, which is about 86% on all the homologous chromosomes.

**Figure 4:**
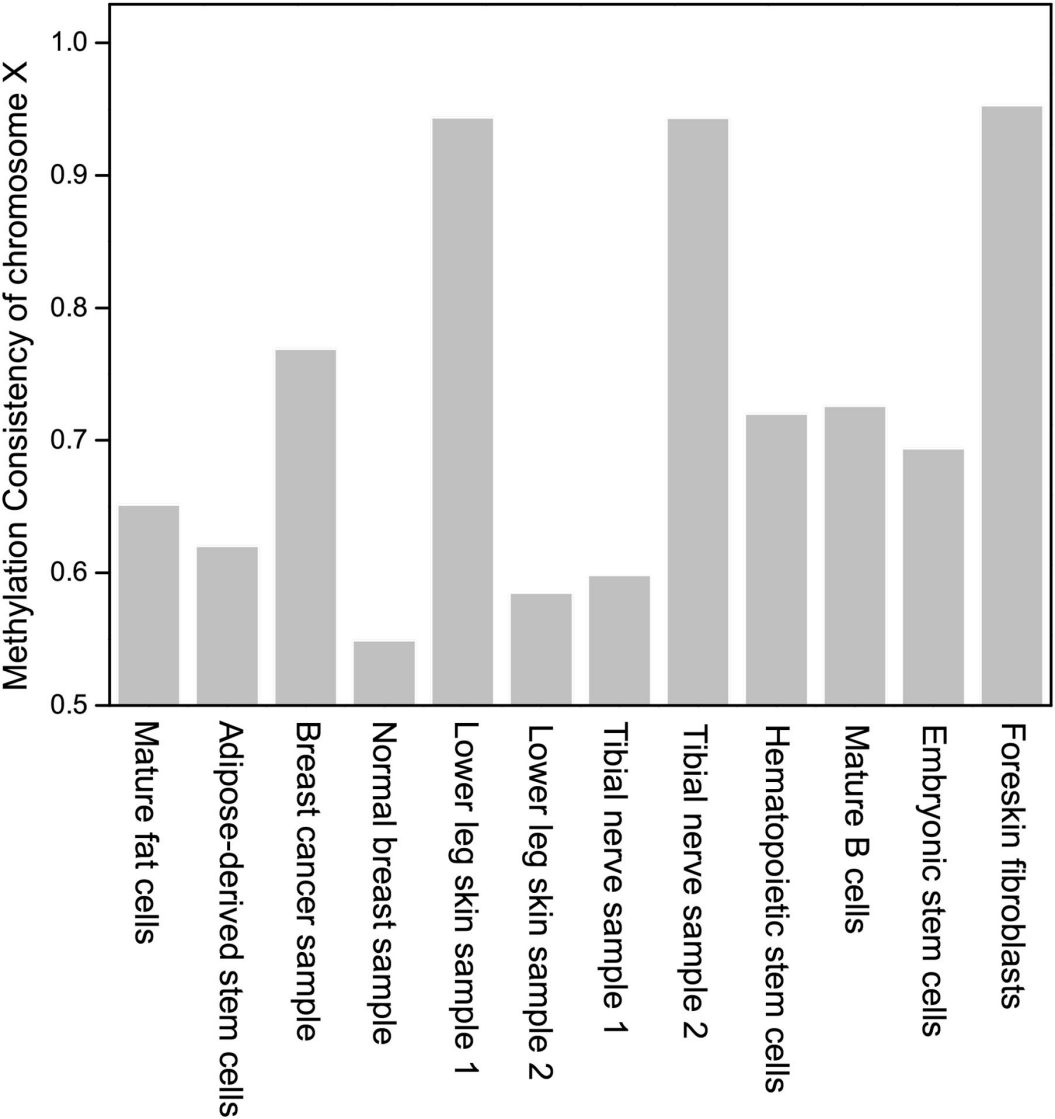
The comparison of methylation consistency of chromosome X in different samples.

The methylation consistency of chromosome X indicates the gender of a sample. In Figure 4, it can be observed that three samples with methylation consistency above 94% are derived from male, while samples with methylation consistency ranging from 54 to 72% are derived from female which is much lower than that of other homologous autosomes. It coincides with the previous studies that the methylation between two homologous chromosome X in female are different, one of which is inactive and highly methylated (Mohandas et al., 1981; Goto and Monk, 1998).

**Figure 5:**
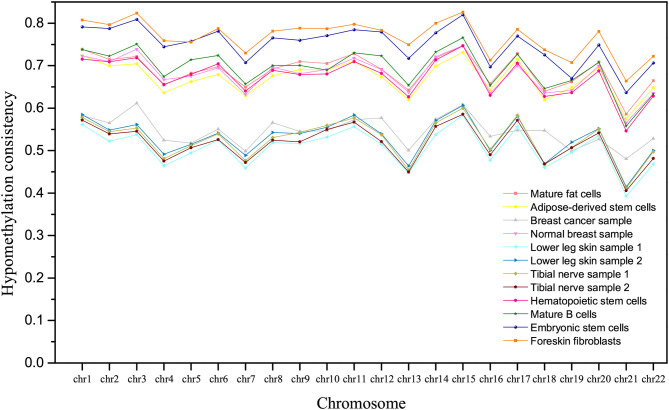
The comparison of hypomethylation consistency of homologous chromosomes in different tissues/cell types.

Further, we compared the hypomethylation consistency in different samples. The hypomethylation consistency between two homologous chromosomes in a sample can be defined as the ratio of the number of CpGs in VMHs with consistent MHM *LL* to that in all VMHs. From Figure 5, we can observe that the hypomethylation consistency of derived cells is higher than that of the corresponding undifferentiated stem cells, which is consistent with the former studies that methylation decrease with the degree of differentiation increased (Laurent et al., 2010). In Figure 5, we can find that the mature fat cells are more hypomethylated than adipose-derived stem cells, mature B cells are more hypomethylated than hematopoietic stem cells, and foreskin fibroblasts are more hypomethylated than embryonic stem cells. It is also noted that the hypomethylation consistency of breast cancer sample is much lower than that of normal breast sample on homologous chromosome.

In addition, it is interesting to observe that the tissues/cell types can be roughly clustered into three groups according to the hypomethylation consistency, as shown in Figure 5. Lower leg skin and tibial nerve have similar hypomethylation consistency and they belong to the ectoderm. The hypomethylation consistency of mature fat cells, adipose-derived stem cells, mature B cells, hematopoietic stem cells and the normal breast sample are similar, and these tissues/cell types belong to the mesoderm. The hESCs and hESC-Fibro cell types have high hypomethylation consistency in chromosomes, which are higher than that of other tissues/cell types.

### 3.2. Identifying DMRs Between Two Samples

MHap_DMR was applied to identify DMRs in four pairs of samples, including breast cancer vs. normal breast, mature fat cells vs. adipose-derived stem cells, embryonic stem cells (hESCs) vs. foreskin fibroblasts (hESC-Fibro cells), and mature B cells vs. hematopoietic stem cells. In this study, MHap_DMR reports the DMRs with *p* < 0.05 and *MHD* > 0.5.

Based on the MHMs of samples on DMRs, the identified DMRs can be further classified into four groups: 1. hypomethylation mode (a MHM containing *L*) vs. non-hypomethylation mode (a MHM not containing *L*); 2. hypomethylation consistent mode *LL* vs. semi-hypomethylation mode (an unconsistent MHM containing *L*); 3. hypermethylation consistent mode *HH* vs. semi-hypermethylation mode (an unconsistent MHM containing *H*); 4. DMR with other modes. The number of these types of DMRs between each pair of samples is listed in Table 3.

**Table 3:** Four types of DMRs identified by MHap_DMR for each pair of samples.

	**Type 1 DMR**	**Type 2 DMR**	**Type 3 DMR**	**Type 4 DMR**
**Pairs of samples**	**(hypo-**	**(consistent hypo-**	**(consistent hyper-**	
	**vs**.	**vs**.	**vs**.	**with**
	**non-hypo)**	**semi-hypo)**	**semi-hyper)**	**other modes**
Mature fat cells	1,156	1,032	583	309
vs.	(LL vs. HH: 1)	(LL vs. HL: 574)		
Adipose-derived stem cells	(HL vs. HH: 459)			
Breast cancer	20,138	1,351	0	0
vs.	(LL vs. HH: 15,175)	(LL vs. HL: 1,351)		
Normal breast	(HL vs. HH: 650)			
Hematopoietic stem cells	1,468	625	182	257
vs.	(LL vs. HH: 391)	(LL vs. HL: 286)		
Mature B cells	(HL vs. HH: 223)			
Embryonic stem cells	6,856	2,812	914	340
vs.	(LL vs. HH: 2,698)	(LL vs. HL: 1,490)		
Foreskin fibroblasts	(HL vs. HH: 1,465)			

To investigate the methylation changing directions at the methylation haplotype level, the number of some subtypes of DMRs in Type 1 and Type 2 DMRs is specified. For example, in Type 1 DMRs, the number of DMRs with hypomethylation consistent mode *LL* vs. hypermethylation consistent mode *HH* and the number of DMRs with hypomethylation unconsistent mode *HL* vs. hypermethylation consistent mode *HH* are listed.

In Type 1 DMRs, it can be observed that there is only 1 DMR with hypomethylation consistent mode *LL* vs. hypermethylation mode consistent *HH* in mature fat cells and adipose-derived stem cells. It may indicate that the methylation statuses of two homologous chromosomes are seldom changed simultaneously during the differentiation from adipose-derived stem cells to mature fat cells.

In Type 1 DMRs between breast cancer and normal breast, it can be observed that there are 13,173 DMRs with hypermethylation consistent mode *HH* in breast cancer and hypomethylation consistent mode *LL* in normal breast, while there are only 2,002 DMRs with hypomethylation consistent mode *LL* in breast cancer and hypermethylation consistent mode *HH* in normal breast. It suggests that many regions with hypomethylation consistent mode *LL* in normal breast become hypermethylated in breast cancer, while a small quantity of regions with hypermethylation consistent mode *HH* in normal breast become hypomethylated in breast cancer. Further, comparing the number of four types of DMRs between breast cancer and normal breast, it may indicate that, in breast cancer, the methylation statuses of homologous chromosomes changes in the same direction (hypomethylated or hypermethylated) simultaneously in many cases. The MHMs of DMRs among different samples can indicate the methylation changing directions of homologous chromosomes in cell differentiation and cancerization.

### 3.3. Compared With Comparative Methods

To further demonstrate the performance of MHap_DMR, four comparative tools were also applied to these four pairs of samples, including CpG_MPs (Su et al., 2012), DMRCaller (Catoni et al., 2018), SMART (Liu et al., 2015), and Metilene (Jühling et al., 2016). The default parameter settings were adopted when running these methods.

The numbers of DMRs identified by different methods are compared, as shown in Table 4. Metilene always predicts a larger number of DMRs with low methylation level differences than other methods. MHap_DMR predicts a smaller number of DMRs than other methods, because it works on candidate regions predefined on the CpG dense regions. All the methods report a largest number of DMRs between breast cancer sample and normal breast sample, and a second largest number of DMRs between embryonic stem cells and foreskin fibroblasts. This consistency indicates that DNA methylation is altered a lot in cancerization, and the methylation difference between embryonic stem cells and foreskin fibroblasts is larger than that between other types of stem cells and the cells derived from these stem cells.

**Table 4:** The number of DMRs identified by different methods.

**Pairs of samples**	**MHap_DMR**	**CpG_MPs**	**DMRCaller**	**SMART**	**Metilene**
Mature fat cells					
vs.	3,080	932	4,081	2,152	44,359
Adipose-derived stem cells					
Breast cancer					
vs.	21,489	233,298	861,108	353,565	357,980
Normal breast					
Hematopoietic stem cells					
vs.	2,532	26,526	172,475	50,453	75,180
Mature B cells					
Embryonic stem cells					
vs.	10,922	130,376	385,877	282,617	338,631
Foreskin fibroblasts					

## 4. Conclusion

In this paper, MHap is developed to construct methylation haplotypes for homologous chromosomes in CpG dense regions. Through the analysis based on methylation haplotypes of homologous chromosomes, we found that majority of methylation haplotypes are consistent between homologous autosomes, while a lower methylation consistency was observed in the breast cancer sample. Further, the hypomethylation consistency of derived cells is higher than that of the corresponding undifferentiated stem cells. The hypomethylation consistency can be used as a feature for cell clustering. DMRs identified by MHap_DMR based on methylation haplotypes can help to investigate the methylation changing directions of homologous chromosomes in cell differentiation and cancerization.

## Data Availability Statement

Publicly available datasets were analyzed in this study. This data can be found at: the ENCODE project (https://www.encodeproject.org/) through the access sample ids ENCSR930WUY, ENCSR128RMY, ENCSR752OCM, and ENCSR658MZU, the GEO database (https://www.ncbi.nlm.nih.gov/geo/) through GEO accession numbers GSE29069, GSE19418, and GSE31971, and the NCBI SRA database (https://www.ncbi.nlm.nih.gov/sra) under the accession number SRA0238292.

## Author Contributions

XP and XD conceived and designed the approach. XP and YL performed the experiments. YL and XK analyzed the data. XP wrote the manuscript. XP and XZ supervised the whole study process and revised the manuscript. All authors have read and approved the final version of manuscript.

## Conflict of Interest

The authors declare that the research was conducted in the absence of any commercial or financial relationships that could be construed as a potential conflict of interest.

## References

[B1] Abante J. Fang Y. Feinberg A. Goutsias J. (2020). Detection of haplotype-dependent allele-specific DNA methylation in WGBS data. Nat. Commun. 11, 1–13. 10.1038/s41467-020-19077-1 33067439 PMC7567826

[B2] Baylin S. B. (2005). DNA methylation and gene silencing in cancer. Nat. Rev. Clin. Oncol. 2:S4. 10.1038/ncponc0354 16341240

[B3] Catoni M. Tsang J. M. Greco A. P. Zabet N. R. (2018). DMRcaller: a versatile R/bioconductor package for detection and visualization of differentially methylated regions in CpG and non-CpG contexts. Nucl. Acids Res. 46:e114. 10.1093/nar/gky602 29986099 PMC6212837

[B4] Chen Q. Lai D. Lan W. Wu X. Chen B. Chen Y.-P. P.. (2019). ILDMSF: inferring associations between long non-coding RNA and disease based on multi-similarity fusion. IEEE/ACM Trans. Comput. Biol. Bioinformatics. 18, 1106–1112 10.1109/TCBB.2019.2936476 31443046

[B5] Cheung W. A. Shao X. Morin A. Siroux V. Kwan T. Ge B.. (2017). Functional variation in allelic methylomes underscores a strong genetic contribution and reveals novel epigenetic alterations in the human epigenome. Genome Biol. 18, 1–21. 10.1186/s13059-017-1173-7 28283040 PMC5346261

[B6] Condon D. E. Tran P. V. Lien Y.-C. Schug J. Georgieff M. K. Simmons R. A.. (2018). Defiant:(dmrs: easy, fast, identification and annotation) identifies differentially methylated regions from iron-deficient rat hippocampus. BMC Bioinformatics 19:31. 10.1186/s12859-018-2037-1 29402210 PMC5800085

[B7] Eden A. Gaudet F. Waghmare A. Jaenisch R. (2003). Chromosomal instability and tumors promoted by DNA hypomethylation. Science 300:455. 10.1126/science.1083557 12702868

[B8] Feng H. Conneely K. N. Wu H. (2014). A Bayesian hierarchical model to detect differentially methylated loci from single nucleotide resolution sequencing data. Nucl. Acids Res. 42:e69. 10.1093/nar/gku154 24561809 PMC4005660

[B9] Gomez L. Odom G. J. Young J. I. Martin E. R. Liu L. Chen X.. (2019). coMethDMR: accurate identification of co-methylated and differentially methylated regions in epigenome-wide association studies with continuous phenotypes. Nucl. Acids Res. 47:e98. 10.1093/nar/gkz590 31291459 PMC6753499

[B10] Goto T. Monk M. (1998). Regulation of x-chromosome inactivation in development in mice and humans. Microbiol. Mol. Biol. Rev. 62, 362–378. 10.1128/MMBR.62.2.362-378.1998 9618446 PMC98919

[B11] Hansen K. D. Langmead B. Irizarry R. A. (2012). BSmooth: from whole genome bisulfite sequencing reads to differentially methylated regions. Genome Biol. 13:R83. 10.1186/gb-2012-13-10-r83 23034175 PMC3491411

[B12] Hebestreit K. Dugas M. Klein H.-U. (2013). Detection of significantly differentially methylated regions in targeted bisulfite sequencing data. Bioinformatics 29, 1647–1653. 10.1093/bioinformatics/btt263 23658421

[B13] Hodges E. Molaro A. Dos Santos C. O. Thekkat P. Song Q. Uren P. J.. (2011). Directional DNA methylation changes and complex intermediate states accompany lineage specificity in the adult hematopoietic compartment. Mol. Cell 44, 17–28. 10.1016/j.molcel.2011.08.026 21924933 PMC3412369

[B14] Hon G. C. Hawkins R. D. Caballero O. L. Lo C. Lister R. Pelizzola M.. (2012). Global DNA hypomethylation coupled to repressive chromatin domain formation and gene silencing in breast cancer. Genome Res. 22, 246–258. 10.1101/gr.125872.111 22156296 PMC3266032

[B15] Hu X. Li M. Wang L. Li X. Wu F.-X. Wang J. (2019). Classification of schizophrenia by iterative random forest feature selection based on DNA methylation array data, in 2019 IEEE International Conference on Bioinformatics and Biomedicine (BIBM) (San Diego, CA), 807–811. 10.1109/BIBM47256.2019.8983308

[B16] Jühling F. Kretzmer H. Bernhart S. H. Otto C. Stadler P. F. Hoffmann S. (2016). metilene: Fast and sensitive calling of differentially methylated regions from bisulfite sequencing data. Genome Res. 26, 256–262. 10.1101/gr.196394.115 26631489 PMC4728377

[B17] Krueger F. Andrews S. R. (2011). Bismark: a flexible aligner and methylation caller for bisulfite-seq applications. Bioinformatics 27, 1571–1572. 10.1093/bioinformatics/btr167 21493656 PMC3102221

[B18] Lan W. Lai D. Chen Q. Wu X. Chen B. Liu J.. (2020). LDICDL: LncRNA-disease association identification based on collaborative deep learning. IEEE/ACM Trans. Comput. Biol. Bioinformatics. 10.1109/TCBB.2020.3034910 33125333

[B19] Laurent L. Wong E. Li G. Huynh T. Tsirigos A. Ong C. T.. (2010). Dynamic changes in the human methylome during differentiation. Genome Res. 20, 320–331. 10.1101/gr.101907.109 20133333 PMC2840979

[B20] Lea A. J. Tung J. Zhou X. (2015). A flexible, efficient binomial mixed model for identifying differential DNA methylation in bisulfite sequencing data. PLoS Genet. 11:e1005650. 10.1371/journal.pgen.1005650 26599596 PMC4657956

[B21] Lister R. Pelizzola M. Kida Y. S. Hawkins R. D. Nery J. R. Hon G.. (2011). Hotspots of aberrant epigenomic reprogramming in human induced pluripotent stem cells. Nature 471:68. 10.1038/nature09798 21289626 PMC3100360

[B22] Liu H. Liu X. Zhang S. Lv J. Li S. Shang S.. (2015). Systematic identification and annotation of human methylation marks based on bisulfite sequencing methylomes reveals distinct roles of cell type-specific hypomethylation in the regulation of cell identity genes. Nucl. Acids Res. 44, 75–94. 10.1093/nar/gkv1332 26635396 PMC4705665

[B23] Lokk K. Modhukur V. Rajashekar B. Märtens K. Mägi R. Kolde R.. (2014). DNA methylome profiling of human tissues identifies global and tissue-specific methylation patterns. Genome Biol. 15:3248. 10.1186/gb-2014-15-4-r54 24690455 PMC4053947

[B24] Mohandas T. Sparkes R. Shapiro L. (1981). Reactivation of an inactive human x chromosome: evidence for x inactivation by DNA methylation. Science 211, 393–396. 10.1126/science.6164095 6164095

[B25] Ni P. Huang N. Zhang Z. Wang D.-P. Liang F. Miao Y.. (2019). DeepSignal: detecting DNA methylation state from nanopore sequencing reads using deep-learning. Bioinformatics 35, 4586–4595. 10.1093/bioinformatics/btz276 30994904

[B26] Park Y. Figueroa M. E. Rozek L. S. Sartor M. A. (2014). Methylsig: a whole genome DNA methylation analysis pipeline. Bioinformatics 30, 2414–2422. 10.1093/bioinformatics/btu339 24836530 PMC4147891

[B27] Park Y. Wu H. (2016). Differential methylation analysis for BS-seq data under general experimental design. Bioinformatics 32, 1446–1453. 10.1093/bioinformatics/btw026 26819470 PMC12157722

[B28] Scott C. A. Duryea J. D. MacKay H. Baker M. S. Laritsky E. Gunasekara C. J.. (2020). Identification of cell type-specific methylation signals in bulk whole genome bisulfite sequencing data. Genome Biol. 21, 1–23. 10.1186/s13059-020-02065-5 32605651 PMC7329512

[B29] Stockwell P. A. Chatterjee A. Rodger E. J. Morison I. M. (2014). DMAP: differential methylation analysis package for RRBS and WGBS data. Bioinformatics 30, 1814–1822. 10.1093/bioinformatics/btu126 24608764

[B30] Su J. Yan H. Wei Y. Liu H. Liu H. Wang F.. (2012). CpG_MPs: identification of CPG methylation patterns of genomic regions from high-throughput bisulfite sequencing data. Nucl. Acids Res. 41:e4. 10.1093/nar/gks829 22941633 PMC3592415

[B31] Sun S. Yu X. (2016). Hmm-fisher: identifying differential methylation using a hidden Markov model and fisher's exact test. Stat. Appl. Genet. Mol. Biol. 15, 55–67. 10.1515/sagmb-2015-0076 26854292

[B32] The ENCODE Project Consortium (2012). An integrated encyclopedia of DNA elements in the human genome. Nature 489:57. 10.1038/nature11247 22955616 PMC3439153

[B33] Wang Z. Li X. Jiang Y. Shao Q. Liu Q. Chen B.. (2015). swDMR: a sliding window approach to identify differentially methylated regions based on whole genome bisulfite sequencing. PLoS ONE 10:e0132866. 10.1371/journal.pone.0132866 26176536 PMC4503785

[B34] Wen Y. Chen F. Zhang Q. Zhuang Y. Li Z. (2016). Detection of differentially methylated regions in whole genome bisulfite sequencing data using local Getis-Ord statistics. Bioinformatics 32, 3396–3404. 10.1093/bioinformatics/btw497 27493194

[B35] Wu H. Xu T. Feng H. Chen L. Li B. Yao B.. (2015). Detection of differentially methylated regions from whole-genome bisulfite sequencing data without replicates. Nucl. Acids res. 43:e141. 10.1093/nar/gkv715 26184873 PMC4666378

[B36] Xiaoqing P. Hong-Dong L. Fang-Xiang W. Jianxin W. (2020). Identifying the tissues-of-origin of circulating cell-free DNAs is a promising way in noninvasive diagnostics. Brief. Bioinformatics 22:bbaa060. 10.1093/bib/bbaa060 32427285

[B37] Xu Z. Xie C. Taylor J. A. Niu L. (2020). ipDMR: identification of differentially methylated regions with interval p-values. Bioinformatics 37, 711–713. 10.1093/bioinformatics/btaa732 32805005 PMC8248314

[B38] Yan C. Duan G. Wu F.-X. Pan Y. Wang J. (2019). BRWMDA: predicting microbe-disease associations based on similarities and bi-random walk on disease and microbe networks. IEEE/ACM Trans. Comput. Biol. Bioinformatics 17, 1595–1604. 10.1109/TCBB.2019.2907626 30932846

[B39] Yan C. Duan G. Wu F.-X. Pan Y. Wang J. (2021). MCHMDA: Predicting microbe-disease associations based on similarities and low-rank matrix completion. IEEE/ACM Trans. Comput. Biol. Bioinformatics 18, 611–620. 10.1109/TCBB.2019.2926716 31295117

[B40] Yan C. Wang J. Ni P. Lan W. Wu F.-X. Pan Y. (2017). DNRLMF-MDA: predicting microRNA-disease associations based on similarities of microRNAs and diseases. IEEE/ACM Trans. Comput. Biol. Bioinformatics 16, 233–243. 10.1109/TCBB.2017.2776101 29990253

[B41] Yan C. Wang J. Wu F.-X. (2018). DWNN-RLS: regularized least squares method for predicting circRNA-disease associations. BMC Bioinformatics 19(Suppl. 19):520. 10.1186/s12859-018-2522-6 30598076 PMC6311892

